# Effect of 5-Aminolevulinic Acid and Sodium Fluorescein on the Extent of Resection in High-Grade Gliomas and Brain Metastasis

**DOI:** 10.3390/cancers14030617

**Published:** 2022-01-26

**Authors:** Lasse Cramer Ahrens, Mathias Green Krabbenhøft, Rasmus Würgler Hansen, Nikola Mikic, Christian Bonde Pedersen, Frantz Rom Poulsen, Anders Rosendal Korshoej

**Affiliations:** 1 Department of Neurosurgery, Aarhus University Hospital, Palle Juul-Jensens Boulevard 165, J618, DK8200 Aarhus, Denmark; mathka@rm.dk (M.G.K.); nikmik@rm.dk (N.M.); 2 Department of Neurosurgery, Odense University Hospital, DK5000 Odense, Denmark; Rasmus.Wurgler.Hansen@rsyd.dk (R.W.H.); christian.bonde@rsyd.dk (C.B.P.); frantz.r.poulsen@rsyd.dk (F.R.P.); 3 Department of Clinical Medicine, Aarhus University, Incuba Skejby, Building 2, Palle Juul-Jensens Boulevard 82, J618, DK8200 Aarhus, Denmark

**Keywords:** fluorescence-guided surgery, 5-ALA, sodium fluorescein, resection, glioblastoma, glioma, brain metastases

## Abstract

**Simple Summary:**

Complete surgical removal of high-grade gliomas (HGG) is known to increase the overall survival and progression-free survival. Several studies have shown that fluorescence-guided surgery with 5-aminolevulinic acid (5-ALA) increases gross total resection considerably compared to white light surgery (65% vs. 36%). Recently, an off-label fluorophore, sodium fluorescein (SF), has become popular in fluorescence-guided surgery due to numerous utility benefits compared to 5-ALA, including lower cost, non-toxicity, easy administration during surgery and a wide indication for other CNS tumors. However, the level of evidence is inferior compared to 5-ALA. We reviewed the latest literature on fluorescence-guided surgery with 5-ALA and SF for brain tumors with emphasis on fluorescence-guided surgery in HGG and brain metastases. Further, we assessed the advantages and disadvantages of both fluorophores and discussed their future perspectives.

**Abstract:**

Surgery is essential in the treatment of high-grade gliomas (HGG) and gross total resection (GTR) is known to increase the overall survival and progression-free survival. Several studies have shown that fluorescence-guided surgery with 5-aminolevulinic acid (5-ALA) increases GTR considerably compared to white light surgery (65% vs. 36%). In recent years, sodium fluorescein (SF) has become an increasingly popular agent for fluorescence-guided surgery due to numerous utility benefits compared to 5-ALA, including lower cost, non-toxicity, easy administration during surgery and a wide indication range covering all contrast-enhancing lesions with disruption of the blood–brain barrier in the CNS. However, currently, SF is an off-label agent and the level of evidence for use in HGG surgery is inferior compared to 5-ALA. Here, we give an update and review the latest literature on fluorescence-guided surgery with 5-ALA and SF for brain tumors with emphasis on fluorescence-guided surgery in HGG and brain metastases. Further, we assess the advantages and disadvantages of both fluorophores and discuss their future perspectives.

## 1. Introduction

**Figure 1:**
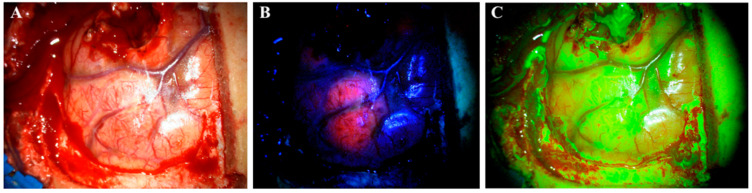
Visualization of a glioblastoma under a surgical microscope with different techniques. (**A**) white light. (**B**) 5-aminolevulinic acid. (**C**) sodium fluorescein.

High-grade gliomas (HGG) are the most common primary malignant brain cancer in adults [1,2]. HGGs arise from astrocytes or oligodendrocytes. They are classified as WHO grade III–IV [3], with an aggressive growth pattern [4,5], a poor prognosis and a median overall survival of approximately 12 to 15 months from diagnosis [6,7]. The current treatment consists of the maximum safe resection followed by chemotherapy and radiotherapy [8]. It is well-documented that the extent of resection (EOR) correlates positively with improved overall survival (OS) and progression-free survival (PFS) [9,10]. Surgery is commonly aimed at gross total resection (GTR) with removal of all contrast-enhancing tumor. However, a recent study by Glenn et al. [11] showed that supramaximal resection (SMR), i.e., resection beyond the boundaries of contrast enhancement, further improved PFS and OS for the 32 glioma patients included in the study compared to GTR and subtotal resection (STR), respectively, with PFS = 15 months vs. 7 months vs. 6 months (*p* < 0.003) and OS = 24 months vs. 11 months vs. 9 months (*p* < 0.004). At the same time, the authors found no statistically significant differences in the postoperative rate of complications between the groups [11]. These findings support the notion that glioma surgery should aim for resection to the functional limit of the peritumoral region. Although an interesting prospect, this concept is associated with technical challenges as access to functional intraoperative mapping techniques is limited in some centers. Furthermore, it can be difficult to distinguish healthy brain tissue from tumor-infiltrated tissue when surgery is performed under white light, which is the most common technique in many centers (Figure 1A). These issues result in suboptimal EOR with historical GTR rates of approximately 36–52% [12,13] despite the use of supportive technologies such as neuronavigation systems and intraoperative MRI.

Recently, fluorescence-guided surgery (FGS) has been becoming increasingly popular among neurosurgeons as a tool to distinguish between pathological and nonpathological tissue intraoperatively [14,15]. The only approved drug for fluorescence-guided glioma surgery is 5-aminolevulinic acid (5-ALA), although sodium fluorescein (SF) has been used increasingly (off-label) by many neurosurgeons due to several benefits. The use of 5-ALA is supported by multiple studies demonstrating superior evidence level compared to SF [16].

The purpose of this review was to elucidate the newest and most essential conclusions about the use of 5-ALA and SF in neurosurgery. We discussed the advantages and disadvantages of both agents and highlighted perspectives for their future use and investigations. We focused on the use of 5-ALA and SF for HGG surgery as this indication currently has the strongest level of evidence, although we also discussed the use of SF for other neurosurgical indications with emphasis on brain metastases. The review primarily included case–control studies.

## 2. Agents for FGS (5-ALA and SF)

Currently, 5-ALA is the only approved drug for FGS. It is administrated orally three hours before surgery in doses of 20 mg/kg [12,13]. However, a recent study indicated that the maximum fluorescence occurs 7–8 h after administration and even later in marginal tumor tissue (8–9 h) [17]. One study even reported fluorescence of 5-ALA 28 h after administration [18]. In addition to glioma surgery, 5-ALA also has other medical applications, such as photodynamic therapy of actinic keratosis, superficial basal cell carcinomas [19,20] and urothelial carcinomas [21]; 5-ALA is a natural precursor molecule to the hemeprotein of hemoglobin synthesis. The compound undergoes a number of intracellular transformations in the cytosol and is transferred to the mitochondria to complete the synthesis. Protoporphyrin IX (PPIX) is the last compound in the synthesis pathway before the enzyme ferrochelatase adds the Fe^2+^ ion and transforms it into a heme. By similar mechanisms, exogenous 5-ALA is taken up by cells and then transformed into PPIX. Due to the downregulated level of ferrochelatase in glioma cells, PPIX accumulates in them instead of in normal cells [22]. PPIX has fluorescent properties and absorbs light in the blue spectrum (375–440 nm) to emit a red–violet light (640–710 nm) upon fluorophore relaxation. This enables neurosurgeons to visualize glioma tissue with accumulated PPIX under a surgical microscope using a red light filter [23] (Figure 1B).

Despite surgical benefits, 5-ALA also has some disadvantages that limit its applications and practical utility. Firstly, the market price of 5-ALA is approximately $2650 per patient. Secondly, 5-ALA must be administered orally 3–4 h before surgery, which can be inconvenient from a logistical and practical point of view and result in a stronger demand for presurgical planning and infrastructure. Thirdly, the compound has phototoxic side effects for 24 h following administration so measures must be taken to avoid exposure of the patient to sunlight or strong white light during this period [14]. Further, intraoperative use of 5-ALA requires total darkening of the operating room, which makes it difficult to simultaneously visualize vessels and other important neuroanatomical structures to be preserved. Inconveniently, it is therefore necessary to switch the light on and off repeatedly during the operation to enforce a safe surgical technique, while benefitting from the fluorescent properties of 5-ALA. Finally, the compound is only reliably absorbed in HGGs and not in other brain tumors or metastases, which limits its applications in general neuro-oncological surgery [24].

These restrictions have resulted in a clinical need for cheaper and less toxic alternatives to 5-ALA, with a wider range of applications and a more convenient administration profile. This has recently led to the rediscovery of SF, which is currently used off-label in some centers around the world. Contrary to 5-ALA, SF is an unspecific fluorescent marker of a disrupted blood–brain barrier (BBB). The mechanism of action for SF is different from 5-ALA as it is not accumulated intracellularly in tumor cells, but rather is distributed and accumulated in the extracellular space throughout brain areas where the BBB is disrupted, e.g., by tumor infiltration [25]. Thereby, SF is not selectively used for HGGs, but rather accumulates in all tumors with contrast enhancement on CT or MR [15]. When first introduced, SF was administrated intravenously in high doses (20 mg/kg) and visualized under a surgical microscope illuminating white light. Recently, the surgical procedure changed and SF is now administrated at the beginning of an operation in low doses of 3–5 mg/kg [15]. SF has the light absorption maximum at 494 nm and emits green fluorescence at 540–690 nm. It is visualized under a surgical microscope with a yellow (560 nm) filter (Figure 1C). SF can be visualized up to 4 h after administration [24]. Although 5-ALA is specific for HGG, SF provides a more distinct border between tumor tissue and brain tissue. Despite the correlation with contrast leakage on CT and MRI, one study found that SF also revealed tumors that were not contrast-enhancing on preoperative MR [26], and so the mechanism of action may be more complex than what is currently understood.

SF has some advantages compared to 5-ALA. The said advantages include (i) being a cheaper alternative (approximately 1/20 the price of 5-ALA); (ii) it has no side effects; (iii) it is easier to use in clinical practice as it is given intravenously at the beginning of an operation and does not require darkening of the operating room during an operation; and lastly, (iv) SF is absorbed nonspecifically where the blood–brain barrier is disrupted, which might make it much more useful for other types of tumors as well [27]. Recent studies, primarily case series with low sample sizes, indicate potential benefits of SF in the surgery of brain metastases [28], meningiomas [29], lymphomas [30,31], pituitary [32,33], pediatric brain stem [34,35], and spinal cord tumors [36,37].

## 3. White Light Surgery Compared to 5-ALA-Guided Surgery in HGGs

Many studies have found increased GTR, OS and PFS with 5-ALA-guided HGG resection compared to conventional white light surgery. Specifically, a recent meta-analysis found GTR rates of 79.1% with 5-ALA use compared to 52.8% without 5-ALA [12]. The OS was increased by approximately three months (95% CI, 2.43–3.68 months; *p* < 0.001), PFS—by approximately one month (95% CI, 0.61–1.45 months; *p* < 0.001) when using 5-ALA compared to conventional surgery. 

In 2006, a pivotal randomized controlled trial (RCT) was performed to evaluate the efficacy of 5-ALA for HGG surgery [13]. The trial included 322 patients with newly diagnosed HGG for whom attempted GTR was deemed feasible. The major exclusion criteria were tumor localization in the midline, basal ganglia, cerebellum, or brain stem, Karnofsky performance score ≤ 60, multifocal disease and competing malignancies. The patients were randomized 1:1 into two arms, i.e., one arm treated with presurgical 5-ALA (20 mg/kg) and fluorescence-guided tumor resection and a control arm operated under conventional white light. All the patients received the same pre- and postoperative treatment, including supportive therapy, concomitant radiochemotherapy and adjuvant chemotherapy. The primary endpoints were GTR and the PFS rate at six months (PFS6). The secondary endpoints were the volume of the residual tumor, OS, neurological deficits and toxic effects. The endpoints were analyzed using the intent to treat. The authors found a significant difference in GTR rates between the groups, with GTR = 65% in the 5-ALA group and 36% in the white light group (*p* < 0.0001). The PFS6 rate was 41% in the 5-ALA group compared to 21% in the control group, respectively (*p* < 0.0003). The OS did not differ significantly between the two groups regardless of patient age. 

In 2014, Diez Valle et al., conducted a retrospective multicenter study including 251 patients from 18 departments [38]. The 5-ALA patients were recruited from highly specialized centers which had 5-ALA equipment available. The controls were recruited from centers without 5-ALA available and operated using white light surgery. The endpoints were GTR (defined as postoperative MRI without contrast enhancement) and PFS6. GTR and PFS6 were significantly improved in the 5-ALA group compared to the controls (67% vs. 45%, *p* < 0.000, and 69% vs. 48%, *p* < 0.002).

Coburger et al. (2015) retrospectively analyzed 66 patients operated in 2012–2014. The inclusion criteria were primary glioblastomas eligible for attempted GTR [39]. They included 33 patients treated with 5-ALA which were matched with 33 patients treated with conventional white light surgery. The groups were matched on MGMT promotor methylation, recurrent surgery, eloquent location, tumor size and age. GTR was defined as resection of over 95% of the tumor on postoperative MRI (<72 h after surgery). GTR and tumor resection (volume) were significantly higher in the 5-ALA patients compared to conventional surgery (99.6% vs. 96%, *p* = 0.004, and 0.1 cc vs. 1.8 cc, *p* = 0.002). Nevertheless, the OS and PFS were not significantly improved.

Della Puppa et al. (2017) retrospectively investigated the effect of carmustine (1,3-bis(2-chloroethyl)-1-nitrosurea, or BCNU)-impregnated wafers (Gliadel^®^) in patients with newly diagnosed glioblastoma [40]. They divided patients into three arms with regard to surgical intervention: group I (BCNU wafers + 5-ALA), group II (5-ALA) and group III (BCNU wafers). GTR was based on the CRET criteria. GTR was 80% (group I), 47% (group II) and 76% (group III). The median OS was 22 months, 18 months and 21 months in groups I, II and III, respectively. All the findings were statistically significant (I vs. II: *p* < 0.0001; I vs. III: *p* = 0.0025, II vs. III: *p* < 0.0001). The median PFS was 11, 10 and 11 months in groups I, II and III, respectively (I vs. II, *p* < 0.0015; I vs. III, *p* = 0.19; II vs. III, *p* < 0.0014). However, this study has several limitations which are crucial for the results. The major problem is that the criteria for BCNU wafers groups are different compared to non-BCNU wafers groups as these tumors are assessed intraoperatively as macroscopic resection > 90%, non-multifocal, noncommunicating with ventricles or crossing corpus callosum. This may explain the difference between groups II and I/III. A summary is shown in Table 1.

## 4. SF-Guided Surgery Compared to White Light Surgery in HGGs

In recent years, SF has become the preferred agent for fluorescence-guided surgery for HGG in some centers, although the evidence is less compared to 5-ALA. Six studies have investigated SF compared to conventional white light surgery [41,42,43,44,45,46], and their conclusions are summarized in the following section.

In 2003, Shinoda et al., evaluated SF surgery in a retrospective study including 105 patients [41]. A total of 32 patients were administrated high-dose SF (20 mg/kg) after durotomy and operated under a surgical microscope with white light illumination. GTR was defined as no contrast-enhancing tumor on postoperative MRI within one month after surgery. The SF group had a significantly higher GTR compared to conventional white light surgery (84.4% vs. 30.1%, *p* = 0.0001); however, there was no significant difference in the OS (15 months vs. 13 months, *p* > 0.05).

Koc et al. (2008) conducted a prospective nonrandomized clinical trial including 80 patients, of which 47 were operated under SF guidance, 33—under white light alone [42]. All the patients were offered open-label SF-guided surgery and the patients who declined the intervention were all treated with conventional white light surgery, making the study prone to selection bias. The SF group was administrated high-dose SF (20 mg/kg). Both groups were operated under the same surgical microscope with white light illumination. GTR was defined as no contrast-enhancing tumor on postoperative MRI (<24 h). The authors found GTR rates of 83% in the SF group and 55% in the white light group (*p* = 0.012), although no differences were observed in the OS (44 weeks vs. 42 weeks, *p* > 0.05). 

Another small nonrandomized prospective study by Chen et al. (2012) included 22 newly diagnosed HGG patients treated with either SF-guided or white light surgery [43]. SF was administrated in doses of 15–20 mg/kg, and the inclusion criteria were comparable to those of Stummer et al. [13]. GTR was defined as no contrast-enhancing tumor on postoperative MRI within seven days after surgery. The authors found a significant difference in GTR rates (80% vs. 33.3%, *p* < 0.047) in favor of SF-guided resection and also demonstrated a significant difference in PFS (7.2 months vs. 5.4 months, *p* < 0.033), respectively [43]. 

In 2017, Catapano et al. (2017) retrospectively analyzed 23 SF-treated patients with newly diagnosed HGG in 2016–2017 [44]. The control group of 25 patients was matched on tumor location, age, gender, etc. The intervention group was administrated SF in doses of 5 mg/kg and operated under an OPMI PENTERO 900 surgical microscope (Carl Zeiss) with an integrated YELLOW 560 filter. GTR was defined as residue < 0.175 cm^3^. GTR rates were significantly higher in the SF group compared to the control group (82.6% vs. 52%, *p* < 0.05). 

Katsevman et al., presented a retrospective study in 2019 including 57 patients with newly diagnosed or recurrent HGG undergoing 64 SF-guided surgeries in 2014–2017 [45]. During that period, the surgical approach with SF changed from a high dose (20 mg/kg) under a normal white light microscope towards a low dose (3–4 mg/kg) administrated at the time of incision intraoperatively and visualized with an OPMI PENTERO 900 surgical microscope (Carl Zeiss) with an integrated YELLOW 560 filter. GTR and STR were defined as ≥ 98% and < 98% volume resection on postoperative MRI (<48 h after surgery). EOR and survival data were compared to a control group of 132 patients undergoing 158 surgeries without SF between 1996 and 2017. SF-guided surgery resulted in a significant increase in GTR compared to white light surgery (73.4% vs. 52.5%, *p* = 0.029), although no significant difference in the OS was detected (78 weeks vs. 60 weeks, *p* = 0.36). Some pitfalls of this study must be taken into consideration. First, the two groups significantly differed in preoperative tumor volume (SF group = 16.8 cm^3^ and control group = 32.5 cm^3^). Second, the study only included EOR data from 40 patients in the control group. Finally, EOR was evaluated by an unblinded assessor. The long inclusion period for controls also questions whether the results are biased by a potential time effect between the groups due to changes in the surgery staff and level of experience, supportive technologies (neuronavigation and intraoperative MRI) and new oncologic treatments [45]. 

Finally, Hong et al. (2019) included 82 patients in 2016–2017 with the same baseline characteristics [46]. The inclusion criteria were HGG and first-time surgery in patients eligible for attempted GTR. The exclusion criteria were severe comorbidity including liver, kidney and heart disease. The patients were divided into two groups operated with either SF guidance or white light alone. GTR was defined as no contrast on postoperative MRI. All the patients were operated under the same surgical microscope OPMI PENTERO 900 (Carl Zeiss). The SF group was administrated SF in low doses (1.5–2 mg/kg) minimum 90 min before durotomy and a YELLOW 560 nm filter was applied to the microscope. The authors reported a GTR rate of 85.7% in the SF-guided group and 62.5% in the control group (*p* = 0.02). 

In summary, several studies report an increased GTR rate in SF-guided tumor resection compared to conventional white light surgery (73.4–85.7% vs. 30.1–62.5%, respectively). However, it is important to note that the level of evidence in the available literature is class III or below as all the studies have important caveats that must be considered, such as lack of randomization and small sample size [47]. An overview of the study conclusions is given in Table 2.

## 5. Direct Comparison of 5-ALA and SF

Until recently, all evidence of SF-guided surgery was based on uncontrolled studies or studies comparing SF to white light surgery alone [41,42,43,44,45,46]. As 5-ALA guidance is a standard practice technology for HGG resection in many centers, studies that directly compare SF with 5-ALA are necessary to investigate the superiority or potential equivalence of either compound based on GTR or survival endpoints. In addition, such studies would also clarify the potential practical and health economic benefits of one agent over the other. In 2019, some of these aspects were investigated in a retrospective cohort study by Hansen et al. [48]. The study included 194 patients diagnosed with new HGG and treated with either 5-ALA or SF in 2012–2017. The major exclusion criteria were non-HGG or inconclusive diagnosis based on final histopathology, biopsy-only surgery, missing pre- and postoperative MRI scans and death within 30 days after surgery. The primary endpoint was the difference in EOR rates measured using the RANO criteria (GTR defined as no tumor residue or non-measurable residue) and volumetrically using the complete resection of contrast-enhancing tumor (CRET) criteria (defined as < 0.175 cm^3^ residual tumor). The secondary endpoints were PFS and OS. The authors found no significant difference in GTR or CRET between 5-ALA and SF (64% vs. 62% (OR, 0.90, *p* = 0.76) and 29.5% vs. 36.2% (OR, 1.34, *p* = 0.86), respectively). Furthermore, no difference in the OS was detected between the two groups (14.75 months and 19.75 months, *p* = 0.06, respectively). These results indicate that SF could potentially be equivalent to 5-ALA with respect to the standard efficacy outcomes, which could make it preferable to 5-ALA in some instances. Despite the relatively large sample size, the study by Hansen et al., has some limitations in addition to the common caveats of retrospective studies. Most importantly, the study was conducted over a period of six years and halfway through, the department changed surgical preference from using 5-ALA to SF due to cost/benefit and utility considerations. This potentially led to time-dependent bias because of potential differences in the experience and composition of the surgical staff as well as changes in active protocols and oncological treatment. Finally, it is possible that 5-ALA was used for selected cases in non-eloquent areas, potentially leading to selection bias.

## 6. SF and 5-ALA in Brain Metastases

Although the evidence of 5-ALA is superior in HGG surgery, it is well-known that it does not have an essential part in the removal of brain metastases. In 2019, Marhold et al., administrated 5-ALA to 157 different brain metastases and found that only 66% of the tumors exhibited visible fluorescence [49]. Hence, 5-ALA is not reliable to use in brain metastases and therefore is not routinely used.

Currently, the strongest evidence for FGS in brain metastases is based on the use of SF. Five clinical studies have investigated SF for this indication with the GTR rate as the primary endpoint [50,51,52,53,54]. Only two studies compared SF directly to white light surgery [53,54], while the others did not include a control population.

Okuda et al. (2010) investigated the effect of high-dose SF (20 mg/kg) administrated after durotomy without a yellow filter on the surgical microscope [50]. They included 36 patients and reported a GTR rate of 86.1% (GTR < 10% residual contrast enhancement). This is higher than the historical data on white light surgery without fluorescence agents [55]. Comparable results were achieved by Schebesch et al. (2015) performing SF-guided surgery (200 mg total dose) in 30 patients with cerebral metastasis [51]. SF was injected before durotomy was performed. The authors found bright fluorescence in 90% of the patients and achieved GTR in 83.3% (GTR defined as no contrast-enhancing residual tumor). Updated results from the same group (2017) showed similar GTR rates (83%) based on an additional 65 patients receiving low-dose SF (5 mg/kg dose) [52]. In this study, the EOR was evaluated by a blinded neuroradiologist and GTR was defined as no residual contrast enhancement on postoperative MRI (<72 h after surgery). In both studies, surgery was performed with an OPMI PENTERO 900 surgical microscope (Carl Zeiss) with an integrated YELLOW 560 filter. Xiao et al. (2018) performed a retrospective case–control study on 38 patients diagnosed with breast cancer brain metastasis [53]. The patients were divided into two groups treated with either SF-guided surgery or white light surgery. SF was administrated in low doses of 5 mg/kg and surgery was performed under an OPMI PENTERO 900 surgical microscope (Carl Zeiss) with an integrated YELLOW 560 filter. The authors found significantly higher GTR rates in the SF group compared to controls (94% vs. 62%, *p* = 0.02), although they were unable to detect a significant difference in the OS (24.1 months vs. 22.8 months, *p* > 0.05). Kofoed et al. (2021) compared in a retrospective study the degree of resection and patient outcomes after neurosurgical treatment with either SF or white light [54]. A total of 117 patients with first-time cerebral metastases were included for analysis (56 with SF and 61 without SF). All the patients were operated under an OPMI PENTERO 900 surgical microscope (Carl Zeiss). The SF group was administrated a dose of 200 mg SF at induction of anesthesia and a YELLOW 5060 filter was applied to the microscope. The EOR was evaluated according to the RANO criteria on postoperative MRI within 72 h after surgery. There was no difference between the two groups (number of metastases, preoperative Karnofsky score, gender, eloquent tumor location, type of primary cancer, supra- vs. infratentorial tumor location and preoperative ASA score). The results showed a statistically higher degree of resection in the SF group with 94% having no or no measurable residual tumor compared to 84% in the non-SF group. Overall, the 1-year survival rate in the SF group was significantly higher (44.6%) compared to the non-SF group (31.1%), without differences in postoperative neurological outcomes.

A summary of the studies is shown in Table 3.

## 7. Discussion

The evidence of FGS has increased gradually since the early 2000s. Studies have demonstrated an improved EOR in patients with HGG operated with 5-ALA guidance. Recently, several centers have also incorporated SF-guided surgery due to practical utility advantages and higher cost effectiveness. Despite some obvious benefits of SF, the choice of the fluorophore should be based on a validated and objective clinical endpoint, such as EOR. 

Based on the current literature, it is difficult to compare 5-ALA to SF as the majority of evidence is based on observational studies without controls or individual comparison of either agent with white light surgery. Additionally, most studies differ in their methodological approach, such as study design, inclusion and exclusion criteria, definitions of endpoints, including EOR. We primarily focused on GTR in case–control studies, and among the studies investigating 5-ALA in HGG surgery, the definition of GTR was defined differently in each study; CRET (*n* = 2), tumor resection > 95% (*n* = 1), no contrast-enhancing tumor (*n* = 1). Likewise, the day of postoperative MRI evaluation ranged between 24 h (*n* = 1), 72 h (*n* = 2) and 28 days (*n* = 1). Only one study was a prospective RCT while three studies were retrospective case–control studies. Similarly, in the studies investigating SF in HGG surgery, there are major variations. With regard to the definition of GTR, four studies defined GTR as no contrast-enhancing tumor, one study—according to the CRET criteria, one study—as tumor resection > 98%. Further, the time of postoperative MRI was different in all the six studies, varying from one day to one month. High doses of SF (15–20 mg/kg) were used in two studies under a surgical microscope without a filter and four studies used low-dose SF (1.5–5 mg/kg) under a surgical microscope equipped with a YELLOW 560 nm filter. Only two studies were prospective nonrandomized while the rest were retrospective case–control studies. Hence, it is difficult to compare the 5-ALA and SF studies. However, all studies found increased GTR in the FGS groups compared to conventional white light surgery. Further studies with the same inclusion criteria, definitions, etc. and a direct comparison of each agent are needed.

Hansen et al. [48] recently provided a direct comparison of the two agents and proposed that SF is a rational and plausible alternative to 5-ALA for HGG surgery as the two techniques resulted in comparable GTR rates (62% vs. 64%, *p* = 0.76, respectively), although the study was not designed or powered to make noninferiority or equivalence conclusions. Future studies are necessary to validate the potential strengths and weaknesses of SF and 5-ALA for HGG, ideally as RCT studies designed as noninferiority or equivalence trials. In this respect, it is important to determine a proper margin of noninferiority/equivalence as SF has a significant economic and utility advantage. Specifically, it is important to discuss what level of differences in clinical endpoint performance (e.g., OR, PFS or EOR) would be acceptable to gain the utility advantages of SF and obtain a suitable cost/benefit balance. 

Furthermore, an important aspect to consider when choosing between SF and 5-ALA for HGG surgery is whether the operated lesion is located in a non-eloquent or an eloquent area. A study by Stummer et al., showed that despite residual fluorescent tissue with 5-ALA, 17 of the 35 operations had GTR on postoperative MRI [56]. This implies that removal of all 5-ALA tissue may lead to more radical resections resulting in complications if the tumor is located in near-eloquent areas, even though it may not be necessary to achieve GTR based on postoperative MRI. SF is shown to be comparable to boundaries on MRI [46]. However, some studies indicate that 5-ALA is able to detect tumor tissue beyond the contrast-enhancing boundaries on MRI which is often used to measure the EOR [57,58,59]. Furthermore, Kiesel et al. (2019) showed that despite the absence of visible fluorescence of 5-ALA, 33 of the 67 biopsies still detected infiltrative tumor tissue [60]. With respect to the two compounds likely performing equally with respect to the standard EOR measures based on contrast enhancement, 5-ALA could be preferable when operating in non-eloquent areas. Furthermore, it might be relevant to use alternative quantifications of EOR, such as volume of the resected tissue, to compare the two agents directly as 5-ALA may in fact provide supramaximal resection while SF likely produces gross total resection. On the other hand, when maximal safe resection of near-eloquent or eloquent lesions is needed, SF might be better than 5-ALA since the boundary between fluorescence and non-contrast-enhancing brain parenchyma is very distinct. 

For hospitals purchasing commercial 5-ALA products, there is a considerable benefit of SF over 5-ALA reflected in the price difference. In Denmark, the current price of 5-ALA is approximately €2300 while that of SF is approximately €42 [61,62]. For hospitals with in-house production of 5-ALA, this difference may be less pronounced. Due to easier preoperative administration and handling, SF might potentially improve surgical planning and capacity management. Furthermore, SF might result in shorter surgeries as resection can be done during fluorescence visualization without the pauses required with 5-ALA. Future studies should ideally include a direct cost/benefit comparison of the two agents.

Several articles [13], including a recent meta-analysis [10], provide solid evidence for the correlation between EOR and clinical outcomes. Interestingly, a majority of studies on FGS have been able to demonstrate a significant benefit in the EOR using this technique (compared to white light surgery), although a significant improvement in the OS has not been shown. This leaves a significant gap in the current evidence for FGS and a need for future high-quality trials to address this aspect.

Regarding the potential benefits and utility of SF for other CNS tumors including brain metastases, there are strong indications that this technique is useful for contrast-enhancing lesions in general, although the level of evidence is low. There is a strong need for high-quality (class I or II) studies to address the relevance of FGS for these broad indications.

To our knowledge, 5-ALA and SF are not specific to any specific molecules or genetic sub-identities [63]. However, if future studies find a correlation between the amount of fluorescence and genetic markers, it will open an opportunity to use personalized medicine, e.g., intraoperative implantation of wafers for targeted drug treatment. Furthermore, integration of artificial intelligence (AI) in FGS is interesting. AI systems that are capable of correlating fluorescent tissue with the probability of cancer tissue could be helpful for neurosurgeons when resecting diffuse marginal tumors. Currently, intraoperative use of hyperspectral imaging (HSI) and Raman scattering microscopy is investigated [64].

In summary, there is a strong need for future studies to elucidate the advantages and disadvantages of 5-ALA and SF in direct comparison for HGG surgery, but also for the use of SF in general. Ideally, comparative studies of 5-ALA and SF for HGG surgery would be designed as noninferiority or equivalence RCTs, although additional high-quality retrospective analyses would also improve the level of evidence. Evaluation of SF for other indications, e.g., brain metastases, should ideally be designed as comparative randomized studies against white light surgery (standard practice). In addition, care must be taken to choose a suitable endpoint for future trials to ensure statistical power. Finally, cost/benefit analyses would be useful to qualify the choice of fluorescent agents for different indications. 

Currently, we are planning two separate trials in a class II setting; one study including a direct comparison of 5-ALA and SF in HGGs and one including SF-guided surgery compared to conventional white light surgery in brain metastases.

## 8. Conclusions

We find that there is generally solid evidence for the use of FGS for multiple indications in neuro-oncology. Although 5-ALA remains the gold standard for FGS in HGG surgery, recent studies have indicated that SF may be a plausible alternative for HGG surgery. Additional comparative studies with 5-ALA and SF are needed to determine if SF is a feasible alternative based on GTR. Furthermore, SF is useful for a multitude of contrast-enhancing CNS tumors, including brain metastases. SF has several benefits over 5-ALA, including a broader spectrum of indications, lower cost and more practical utility features. 

## Figures and Tables

**Table 1:** HGG studies with 5-ALA-guided surgery compared to white light surgery.

Study	Patients (N)	Study Design	Definitions (GTR and Postoperative MRI after Surgery)	Results
Stummer et al. (2006)	322	Prospective randomized controlled trial	GTR = residual tumor < 0.175 cm^3^ Postoperative MRI: <72 h	GTR: 65% vs. 36% (*p* < 0.0001) * PFS6: 41% vs. 21.1% (*p* < 0.0003) * OS: 15.2 months vs. 13.5 months (*p* < 0.1)
Diez Valle et al. (2014)	251	Retrospective case–control study	GTR = no contrast-enhancing tumor Postoperative MRI: <28 days	GTR: 67% vs. 45% (*p* < 0.000) * PFS6: 69% vs. 48% (*p* < 0.002) *
Coburger et al. (2015)	66	Retrospective case–control study	GTR ≥ 95% tumor resection Postoperative MRI: <72 h after surgery	GTR: 99.6% vs. 96.0% (*p* < 0.004) * Volume: 0.1 cc vs. 1.8 cc (*p* < 0.02) * OS: 18 months vs. 17 months (*p* < 0.708) PFS: 6 months vs. 6 months (*p* < 0.309)
Della Puppa et al. (2017)	122	Retrospective case–control study	GTR = residual tumor < 0.175 cm^3^ Postoperative MRI: <24 h	GTR: 80% (BCNU wafers + 5-ALA) 47% (5-ALA) 76% (BCNU wafers) OS: 22 months (BCNU wafers + 5-ALA) 18 months (5-ALA) 21 months (BCNU wafers) PFS: 11 months (BCNU wafers + 5-ALA) 10 months (5-ALA) 11 months (BCNU wafers)

Note: * statistically significant *p*-value < 0.05. 5-ALA = 5-aminolevulinic acid, BCNU = carmustine (1,3-bis(2-chloroethyl)-1-nitrosurea, GTR = gross total resection, N = number, OS = overall survival, PFS = progression-free survival.

**Table 2:** HGG studies with SF-guided surgery compared to white light surgery.

Study	Patients (N)	Study Design	SF Dose	Yellow 560 nm Filter	Definitions (GTR and Postoperative MRI after Surgery)	Results
Shinoda et al. (2003)	105	Retrospective case–control study	20 mg/kg	None	GTR = no contrast-enhancing tumor Postoperative MRI: 1 month	GTR: 84.4% vs. 30.1% (*p* = 0.0001) OS: 15 months vs. 13 months (*p* > 0.05)
Koc et al. (2008)	70	Nonrandomized prospective study	20 mg/kg	None	GTR = no contrast-enhancing tumor Postoperative MRI: <24 h	GTR: 83% vs. 55% (*p* = 0.012) * OS: 44 weeks vs. 42 weeks (*p* > 0.05)
Chen et al. (2012)	22	Nonrandomized prospective study	15–20 mg/kg	None	GTR = no contrast-enhancing tumor Postoperative MRI: <7 days	GTR: 80% vs. 33.3% (*p* < 0.047) * PFS: 7.2 months vs. 5.4 months (*p* < 0.033) *
Catapano et al. (2017)	48	Retrospective case–control study	5 mg/kg	Yes	GTR = residual tumor < 0.17 cm^3^ Postoperative MRI: <72 h	GTR: 82.6% vs. 52% (*p* < 0.05) *
Katsevman et al. (2019)	189	Retrospective case–control study	3–3 mg/kg	Yes	GTR = tumor resection > 98% Postoperative MRI: <48 h	GTR: 73.4% vs. 52.5% (*p* = 0.029) * OS: 78 weeks vs. 60 weeks (*p* = 0.36)
Hong et al. (2019)	82	Case-control study	1.5–2 mg/kg	Yes	GTR = no contrast-enhancing tumor Postoperative MRI: not stated	GTR: 85.7% vs. 62.5% (*p* = 0.02) *

Note: * statistically significant *p*-value (<0.05). GTR = gross total resection, N = number, OS = overall survival, PFS = progression-free survival, SF = sodium fluorescein.

**Table 3:** Overview of studies with SF in brain metastases.

Study	Patients (N)	Study Design	SF Dose	Yellow 560 nm Filter	Definitions (GTR and Postoperative MRI after Surgery)	Results
Okuda et al. (2010)	36	Retrospective observational study	20 mg/kg	None	GTR = total resection Postoperative MRI: not stated	GTR: 86.1%
Schebesch et al. (2015)	30	Retrospective observational study	200 mg total	None	GTR = no contrast-enhancing tumor Postoperative MRI: <48 h	GTR: 83.3%
Hohne et al. (2017)	95	Retrospective observational study	200 mg total (*n* = 30) 5 mg/kg (*n* = 65)	None (*n* = 30) Yes (*n* = 65)	GTR = no contrast-enhancing tumor Postoperative MRI: <72 h	GTR: 83%
Xiao et al. (2018)	38	Retrospective case–control study	5 mg/kg	Yes	GTR = no contrast-enhancing tumor Postoperative MRI: <72 h	GTR: 94% (SF) vs. 62% (white light) (*p* = 0.02) * OS: 24.1 months vs. 22.8 months (*p* > 0.05)
Kofoed et al. (2021)	117	Retrospective case–control study	200 mg total	Yes	GTR ≤ 10 mm residual tumor Postoperative MRI: <72 h	GTR: 94% vs. 84% (*p* = 0.000) * OS (1 year): 44.6% vs. 31.1% (*p* = 0.0001) *

Note: * statistically significant *p*-value (<0.05). GTR = gross total resection, N = number, OS = overall survival, SF = sodium fluorescein.
